# New Approaches to Tay-Sachs Disease Therapy

**DOI:** 10.3389/fphys.2018.01663

**Published:** 2018-11-20

**Authors:** Valeriya V. Solovyeva, Alisa A. Shaimardanova, Daria S. Chulpanova, Kristina V. Kitaeva, Lisa Chakrabarti, Albert A. Rizvanov

**Affiliations:** ^1^Institute of Fundamental Medicine and Biology, Kazan Federal University, Kazan, Russia; ^2^School of Veterinary Medicine and Science, University of Nottingham, Nottingham, United Kingdom

**Keywords:** lysosomal storage diseases, GM2-gangliosidosis, β-hexosaminidase, Tay-Sachs disease, neurodegeneration, inflammation, gene therapy, bone marrow transplantation

## Abstract

Tay-Sachs disease belongs to the group of autosomal-recessive lysosomal storage metabolic disorders. This disease is caused by β-hexosaminidase A (HexA) enzyme deficiency due to various mutations in α-subunit gene of this enzyme, resulting in GM2 ganglioside accumulation predominantly in lysosomes of nerve cells. Tay-Sachs disease is characterized by acute neurodegeneration preceded by activated microglia expansion, macrophage and astrocyte activation along with inflammatory mediator production. In most cases, the disease manifests itself during infancy, the “infantile form,” which characterizes the most severe disorders of the nervous system. The juvenile form, the symptoms of which appear in adolescence, and the most rare form with late onset of symptoms in adulthood are also described. The typical features of Tay-Sachs disease are muscle weakness, ataxia, speech, and mental disorders. Clinical symptom severity depends on residual HexA enzymatic activity associated with some mutations. Currently, Tay-Sachs disease treatment is based on symptom relief and, in case of the late-onset form, on the delay of progression. There are also clinical reports of substrate reduction therapy using miglustat and bone marrow or hematopoietic stem cell transplantation. At the development stage there are methods of Tay-Sachs disease gene therapy using adeno- or adeno-associated viruses as vectors for the delivery of cDNA encoding α and β HexA subunit genes. Effectiveness of this approach is evaluated in α or β HexA subunit defective model mice or Jacob sheep, in which Tay-Sachs disease arises spontaneously and is characterized by the same pathological features as in humans. This review discusses the possibilities of new therapeutic strategies in Tay-Sachs disease therapy aimed at preventing neurodegeneration and neuroinflammation.

## Introduction

GM2-gangliosidoses are a group of autosomal-recessive lysosomal storage disorders (LSDs). These diseases result from a deficiency of lysosomal enzyme β-hexosaminidase (Hex), which is responsible for GM2 ganglioside degradation (Ferreira and Gahl, 2017). Gangliosides are the main glycolipids of neuronal cell plasma membranes which ensure normal cellular activities (Sandhoff and Harzer, 2013). There are two major β-hexosaminidase isoenzymes: HexA consists of two subunits, α and β; HexB is a homodimer consisting of two β-subunits (Ferreira and Gahl, 2017). The two subunits of HexA enzyme, α and β, are synthesized at the endoplasmic reticulum (ER) where glycosylation, the formation of intramolecular disulfide bonds and dimerization take place (Weitz and Proia, 1992; Maier et al., 2003). Besides HexA and HexB isoenzymes a homodimer consisting of two α-subunits, called HexS, is also found (Hou et al., 1996).

After the dimerization of subunits in ER, β-hexosaminidase is transported to the Golgi complex, where it undergoes post-translational modification. The most important of these is the addition of mannose-6-phosphate (M6P) to the side chains of the oligosaccharide (Sonderfeld-Fresko and Proia, 1989). The residues of phosphorylated mannose can be considered as an address mark recognized by specific receptors found on the inner surface of the Golgi complex’s membranes. With the aid of this mark a lysosome recognizes the enzyme and absorbs it (Weitz and Proia, 1992).

**FIGURE 1:**
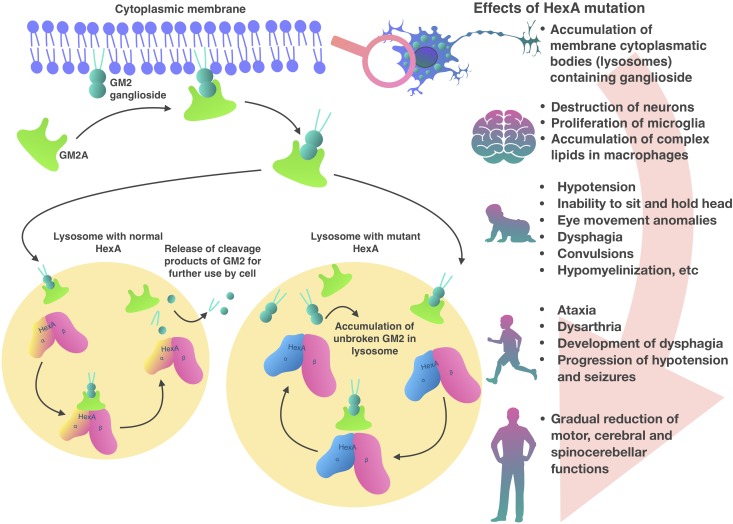
Pathogenesis of Tay-Sachs disease.

Inside lysosomes α and β subunits are proteolytically processed into a mature form (Hubbes et al., 1989). Also in the lysosome, the presentation of the GM2 ganglioside substrate from the bilayer to the HexA active site requires the presence of GM2 activator protein (GM2A) (Martino et al., 2002b; Cachon-Gonzalez et al., 2006). GM2A a co-factor and is necessary in order to make lipophilic GM2 ganglioside available for hydrolysis in the hydrophilic medium of the lysosome (Renaud and Brodsky, 2016; Sandhoff, 2016; Figure 1). HexA and HexB can hydrolyze a wide range of substrates with terminal *N*-acetylglucosamine residues (GlcNAc) to β-bonds. Only the HexA isoenzyme can interact with a GM2 ganglioside-GM2A complex (Lemieux et al., 2006). Although only HexA hydrolyzes GM2 ganglioside, both isoenzymes can hydrolyze glycoproteins, glycosaminoglycans, and glycolipids (Ferreira and Gahl, 2017).

HexA, HexB, and HexS in the absence of GM2A can also hydrolyze synthetic substrates, for example, 4-methylumbelliferone-GlcNAc fluorescent substrate (4MUG). Another compound related to 4MUG is 4-methylumbelliferyl-GlcNAc-6-sulfate (4MUGS) which is only hydrolyzed by isoenzymes HexA and HexS. These compounds are used in GM2-gangliosidoses diagnosis and detection of *HEXA* and *HEXB* gene mutation carriers (Cachon-Gonzalez et al., 2012).

The HexA enzyme is a product of *HEXA* and *HEXB* genes that encode, respectively, the α and β subunits, the amino acid sequence identity of which is about 60% (Mark et al., 2003; Dersh et al., 2016).

## Pathogenesis of GM2-Gangliosidosis

GM2-gangliosidosis can be caused by mutations in three genes: *HEXA* (15th chromosome), *HEXB* (5th chromosome), and *GM2A* (5th chromosome) (Mahuran, 1999; Ferreira and Gahl, 2017). GM2-gangliosidosis includes (I) Tay-Sachs disease (TSD, OMIM 272800), at which mutations occur in the *HEXA* gene and only HexA activity is disrupted (variant B); (II) Sandhoff disease (SD; OMIM 268800), caused by mutations in *HEXB* gene, at which the activity of HexA and HexB is disrupted (variant O); and (III) GM2 activator protein deficiency (OMIM 272750), at which mutations take place in the *GM2A* gene (variant AB) (Mahuran, 1999).

In patients with HexA deficiency GM2 ganglioside accumulates inside lysosomes, which form characteristic inclusions within the cells, so called membranous cytoplasmic bodies, which are enlarged lysosomes filled with gangliosides (Ferreira and Gahl, 2017; Figure 1). The highest concentration of GM2 ganglioside is found in neuronal cells, therefore, the HexA deficiency primarily affects the nervous system, causing mental and motor developmental delay in patients (Myerowitz, 1997). Later, progressive destruction of neurons, proliferation of microglia and accumulation of complex lipids in macrophages are observed in the brain tissue. A similar process develops in the neurons of the cerebellum, basal ganglia, brain stem, spinal cord, spinal ganglia, and also in neurons of the autonomic nervous system. Ganglion cells in the retina also swell and contain GM2 gangliosides, particularly, along the edges of the macula. As a result, a cherry red spot appears in the macula and emphasizes the normal color of the actual choroid, contrasting with the pale, swollen ganglion cells in the affected part of the retina (Ferreira and Gahl, 2017).

An inflammatory response is also observed in patients with GM2-gangliosidosis. Wada et al. (2000) offered a model of acute neurodegeneration in GM2-gangliosidosis and showed that massive death of neurons is preceded by activated microglia expansion. The activation of macrophages and astrocytes along with inflammatory mediators production is also observed (Myerowitz et al., 2002). This inflammatory response can occur before the clinical manifestation of symptoms and aggravates the neurological dysfunction (Wu and Proia, 2004).

In the CNS of GM2-gangliosidosis mouse model, microglial cell activation, and infiltration of inflammatory cells are also observed (Jeyakumar et al., 2003). Hayase et al. (2010) showed that TSD patient cerebrospinal fluid has significantly increased levels of TNF-α pro-inflammatory cytokine, which is involved in the induction of inflammatory response. The authors suggested that an increase in TNF-α level indicates inflammation in the CNS and may contribute to disease progression (Hayase et al., 2010). Utz et al. (2015) identified five possible inflammatory biomarkers ENA-78, MCP-1, MIP-1α, MIP-1β, and TNFR2, increased levels of these in the cerebrospinal fluid is associated with infantile gangliosidosis.

## Tay-Sachs Disease

Tay-Sachs disease is caused by mutations in the *HEXA* gene. The incidence of this disease is one in 100,000 live births (carrier frequency of about one in 250) (Lew et al., 2015). TSD, SD, and GM2A deficiency are clinically similar (Seyrantepe et al., 2018). More than 130 different mutations in *HEXA* gene are described (partial deletion, splicing mutations, nonsense mutations, missense mutations) leading to disruption of transcription, translation, folding, dimerization of monomers and catalytic dysfunction of HexA protein (Myerowitz, 1997; Sakuraba et al., 2006; Mistri et al., 2012). TSD heterogeneity in severity of clinical symptoms and the age at disease onset is determined by residual HexA enzymatic activity that occurs with some mutations (Kaback and Desnick, 1993). Only 10–15% of HexA activity is required in order to prevent the accumulation of GM2 ganglioside (Osher et al., 2015). The three different forms of TSD are classified by severity of clinical symptoms and the age of onset (Patterson, 2013).

Clinical symptoms and course of the infantile form of TSD, which occurs more often than others, are the most studied. The infantile form, which is characterized by onset around 6 months of age and very low HexA activity levels (<0.5%), rapidly manifests with mental and motor developmental delay (Sandhoff and Christomanou, 1979). In the most severe infantile forms symptoms may occur a few months after childbirth (Nestrasil et al., 2018). Common neurodegenerative symptoms in infants are hypotension, inability to sit or hold their head unsupported, eye movement abnormalities, dysphagia, spasms, and hypomyelination (Jarnes Utz et al., 2017). Most patients with the infantile TSD do not survive past 4 years of age (Bley et al., 2011).

The juvenile form of the disease strikes in early childhood, usually at the age of 3–10 years. Common symptoms are ataxia, dysarthria, dysphagia development, hypotension, and spasm progression. Most patients do not live past the age of 15 years (Nestrasil et al., 2018). In adolescent patients the disease is less severe but has a wider range of symptoms (Regier et al., 2016). Common symptoms are limb muscle weakness and abnormal gait which are observed in 88% of patients (Maegawa et al., 2006).

In contrast to the infantile and juvenile forms, the later-onset form is less aggressive, characterized by small mutations and higher residual activity level of HexA, which is 5–20% of normal activity. Symptoms typically appear in adolescence or early adulthood, but can appear later (20–30 years) (Sandhoff and Christomanou, 1979). Therefore, neurodegeneration progression is slowed presenting with delayed onset and gradual decline in motor, cerebral and spinocerebellar function (Deik and Saunders-Pullman, 2014; Osher et al., 2015).

Currently there are several therapy approaches to these diseases, including substrate reduction (Bembi et al., 2006), bone marrow transplantation (Jacobs et al., 2005), hematopoietic (Stepien et al., 2017) or neural stem cell transplantation (Gonzalez et al., 2016), use of anti-inflammatory drugs (Hayase et al., 2010), administration of purified enzyme (Tsuji et al., 2011), and gene therapy to restore expression of a dysfunctional protein (Cachon-Gonzalez et al., 2012).

## TSD Models

To investigate the efficacy of new TSD therapies Liu and Zhao (2016) proposed an *in vitro* model of induced pluripotent stem cells (iPSCs) derived from infantile TSD patient fibroblasts. These cells expressed OCT4, SOX2, NANOG, Tra-1-60, and alkaline phosphatase pluripotency factors and had the ability to differentiate into tissues from all three germ layers (Liu and Zhao, 2016).

The main *in vivo* models of TSD include mice and sheep. The first mouse TSD model was created in 1995 by knockout of the *HEXA* gene. This line of mice lacked HexA activity, however, GM2 ganglioside accumulation and membrane cytoplasmic body formation in neurons occurred only in certain regions of the brain, excluding the olfactory bulb, the cerebral cortex and the anterior horn of the brain. Also, *HEXA* knockout mice had a normal lifespan and no clinical symptoms of TSD (Taniike et al., 1995). A number of other studies show that *HEXA*-defective mice exhibit biochemical and pathological features of TSD without obvious neurological dysfunction (Cohen-Tannoudji et al., 1995; Sango et al., 1995). The difference in the distribution of neuronal storage delineates a difference in ganglioside metabolism between humans and mice. It was shown that mice have one or more sialidases that remove sialic acid from GM2 ganglioside, which can later be hydrolyzed by HexB in *HEXA*-deficient TSD model mice (Yuziuk et al., 1998; Seyrantepe et al., 2018).

In contrast to *HEXA*-deficient mice *HEXB*-deficient TSD mice, develop CNS neurodegeneration, with spasticity, muscle weakness, rigidity, tremor, and ataxia (Sango et al., 1995; Phaneuf et al., 1996). Thus *HEXB*-deficient mice can be useful for the initial evaluation of potential GM2-gangliosidosis treatment.

A strain of mice deficient in *HEXA* and sialidase *NEU3* genes have been developed with a lifespan of 1.5–4.5 months (Seyrantepe et al., 2018). An abnormal accumulation of GM2 ganglioside in the brains of these mice and the presence of membrane cytoplasmic bodies in neurons were found. HEXA/NEU3-deficient mice have progressive neurodegeneration, bone structure anomalies, and neurologic abnormalities such as ataxia, tremor and slow movement. The described pathologies and symptoms in HEXA/NEU3-deficient mice mimic those observed in patients with early onset TSD. This strain of mouse is a suitable model to investigate new TSD therapies (Seyrantepe et al., 2018).

Tay-Sachs disease is also described in other animal species, for example flamingo *Phoenicopterus ruber* (Zeng et al., 2008) and Jacob sheep (Torres et al., 2010). In these species TSD develops spontaneously and is characterized by HexA enzymatic activity deficiency and GM2 ganglioside accumulation (Lawson and Martin, 2016). It was shown that the nucleotide and amino acid sequences identity of the coding region of *HEXA* gene in flamingo and humans is about 70% (Zeng et al., 2008). However, a serious interspecies difference limits the use of flamingos as a model to investigate pathogenesis and therapy of human TSD. In terms of research, one of the most useful species with spontaneously developing TSD is a Jacob sheep. Torres et al. (2010) showed that in Jacob sheep, TSD clinical manifestations are closest to the pathological features in humans. Sheep with TSD also suffered from ataxia, proprioceptive defects and cortical blindness (Porter et al., 2011). Genetic studies showed that HexA activity deficiency in these sheep is associated with a single nucleotide substitution in exon 11 of the *HEXA* gene, which leads to glycine-to-arginine substitution (Torres et al., 2010).

## Substrate Reduction Therapy

Substrate reduction therapy (SRT) utilizes small molecules to slow the rate of glycolipid biosynthesis (Platt et al., 2003). Efficacy of miglustat (N-butyldeoxynojirimycin, NB-DNJ) in the prevention of GM2 ganglioside accumulation was demonstrated in TSD murine models (Platt et al., 1997; Bembi et al., 2006). NB-DNJ is a small iminosugar competitive inhibitor of glucosylceramide synthase. It catalyzes the first committed step of glycosphingolipid synthesis and can penetrate the blood-brain barrier (Boomkamp et al., 2010). It has been shown that that NB-DNJ added to the food of TSD model mice reduces GM2 ganglioside accumulation in the brain by 50% (Platt et al., 1997). Bembi et al. (2006) assessed the clinical efficacy of NB-DNJ in two patients with TSD infantile form. The authors observed a significant concentration of NB-DNJ in the cerebrospinal fluid of the patients. The use of SRT did not stop the neurologic dysfunction progression in patients, however, the authors recommend the use of NB-DNJ for macrocephaly prevention (Bembi et al., 2006). Similar results were described in a clinical trial (NCT00672022) in five patients (Maegawa et al., 2009).

## Enzyme Replacement Therapy

The development of enzyme replacement therapy (ERT) is a promising option for the treatment of lysosomal storage diseases. After ERT therapy many somatic symptoms are decreased, but it is less effective in preventing CNS neurodegeneration since intravenous administration doesn’t allow the enzyme molecule to cross the blood-brain barrier (Jakobkiewicz-Banecka et al., 2007; Sorrentino et al., 2013). ERT is clinically approved for the following diseases: Gaucher disease (Barton et al., 1991; Connock et al., 2006), Fabry disease (Eng et al., 2001), Pompe disease (Klinge et al., 2005), and mucopolysaccharidosis type I (Wraith et al., 2004), type II (Muenzer et al., 2002) and type VI (Harmatz et al., 2004).

The major challenge in creating HexA-based ERT is the need to synthesize both of the enzyme subunits (Tropak et al., 2016). Methylotrophic yeast *Ogataea minuta (Om)* culture, simultaneously expressing the *HEXA* and *HEXB* genes, can be used for the production of recombinant HexA enzymes. The purified HexA was treated with α-mannosidase to expose mannose-6-phosphate (M6P) residues on the N-glycans (Akeboshi et al., 2007; Tsuji et al., 2011). The therapeutic efficacy of recombinant HexA was demonstrated in the SD mouse model (hexb^-/-^ mice) and improvement of motor function, increase of survival rate and inhibition of the induction of MIP-1α were noted (Tsuji et al., 2011). Tropak et al. (2016) created a hybrid μ subunit combining the critical characteristics of α and β HexA subunits. The hybrid μ subunit contains an active α subunit site, a stable β subunit interface, and unique regions of each subunit necessary for interaction with GM2A. To purify the HexM μ-homodimer HEK239 cells with CRISPR deleted *HEXA* and *HEXB* genes and also stably expressing the μ subunit were used. The authors showed that, in combination with GM2A, HexM hydrolyzes the derivative of GM2-ganglioside both *in cellulo* and *in vitro* (Tropak et al., 2016).

Matsuoka et al. (2011) modified the nucleotide sequence of the human *HEXB* gene encoding the chimeric β subunit to contain the partial amino acid sequence of the α subunit. Chinese hamster ovary (CHO) cell lines were modified with the chimeric *HEXB* gene to obtain a cell line with the chimeric HexB stable expression. It was shown that chimeric HexB can degrade artificial anionic substrates and GM2 ganglioside *in vitro*, and also maintain the thermal stability of the wild-type HexB enzyme in plasma. In TSD patient derived fibroblasts it was shown that the treatment of cells with the chimeric HexB led to the incorporation of the enzyme into the cells and the degradation of the accumulated GM2-ganglioside. Intracerebroventricular administration of chimeric HexB to SD model mice restored Hex activity in the brain and reduced the accumulation of GM2-ganglioside in the parenchyma (Matsuoka et al., 2011).

## Bone Marrow Transplantation

Jacobs et al. (2005) published the clinical case of the application of bone marrow transplantation (BMT) with the following substrate reduction therapy to treat the patient with TSD. The use of BMT and Zavesca^®^ (miglustat) led to an increase in HexA activity in leukocytes and plasma 23 months after transplantation, but did not prevent the development of neurological dysfunction.

A case of BMT from a HLA-matched sibling to a 15-year-old patient with late-onset TSD has been described where 8 years after BMT complete graft retention remained unchanged. HexA activity in leukocytes was 187 nmol/mg/h, which is comparable to the enzyme activity in control group leukocytes. HexA activity in plasma was 15 nmol/mg/h, which is three times lower than the lower limit of HexA normal activity (50–250 nmol/mg/h). There was also no intentional tremor progression after BMT (Stepien et al., 2017).

There are cases of BMT in *in vivo* studies on SD mouse models which showed that BMT prolongs the survival rate (from 4.5 to 8 months) (Norflus et al., 1998) and improves neurologic manifestations in laboratory animals (Wada et al., 2000).

An alternative approach for patients who do not have a suitable bone marrow donor is transplantation of hematopoietic stem cells from umbilical cord blood obtained from partially HLA-matched unrelated donors (Martin et al., 2006). Human umbilical cord blood is an important source of stem cells and progenitor cells capable of providing neuroprotective effect in degenerative disease caused by various factors. Transplantation of umbilical cord blood cells is considered to be a promising approach to treat neurodegenerative disease in ischemic or traumatic spinal cord injury (Galieva et al., 2017).

## Gene Therapy

Attempts to correct mutations in *HEXA* gene by gene and cell engineering began in the mid-1990s. The first vectors for the delivery of wild-type *HEXA* gene were adenoviruses. Akli et al. (1996) first produced TSD patient skin-derived fibroblasts expressing the *HEXA* gene by adenovirus transduction. The enzyme activity in the transduced fibroblasts was 40–84% of the norm. The secretion level of an enzyme α-subunit was 25 times higher than the patient’s untreated control fibroblasts (Akli et al., 1996).

*In vivo* studies with HEXA knockout mice showed that in case of intravenous co-administration, *HEXA* and *HEXB* gene expressing adenoviral vectors preferentially transduced liver cells. Delivery of both HexA subunits promoted enzyme secretion in the serum, as well as the enzymatic activity restoration in peripheral tissues (Guidotti et al., 1999). The main limiting factor for successful use of adenoviral vectors in CNS disease treatment is the inability to overcome the blood-brain barrier (Gray et al., 2010). Another disadvantage is the immunogenicity of adenoviral vectors and their tropism to liver cells which leads to a high accumulation of the vector and overexpression of the transgene in this organ which risks development of hepatocellular carcinoma (Nakamura et al., 2003).

Guidotti et al. (1998) also constructed a retroviral vector encoding the human *HEXA* gene cDNA and produced a stable line of hexa^-/-^ mouse fibroblasts with overexpression of the human *HEXA* gene. The resulting fibroblast line secreted the interspecies HexA enzyme: human α-subunit and mouse β-subunit. The cultivation of fibroblasts with HexA deficiency in the culture medium from transduced fibroblasts resulted in restoration of intracellular HexA activity in non-transduced cells. Absorption of the enzyme by non-transduced fibroblasts from the culture medium was the result of receptor-mediated transfer analagous to lysosomal uptake of the enzyme. Thus HexA can pass from the overexpressing cell to neighboring cells that have a receptor essential for recognizing of M6P (Guidotti et al., 1998). This method based on the ability of non-transduced cells to take up the enzyme is termed cross-correction.

The possibility of cross-correction to restore the metabolism of gangliosides in TSD patient-derived fibroblasts *in vitro* has been investigated. A stable NIH3T3 mouse fibroblast cell line with *HEXA* gene overexpression was made using retroviral transduction. It was shown that when cultured in transduced NIH3T3 cells conditioned medium TSD-fibroblasts absorbed a large amount of soluble enzyme, however, there was not a sufficient amount of the enzyme necessary for the degradation of GM2 ganglioside in lysosomes (Martino et al., 2002b). Therefore, the cross-correction-based delivery of HexA is insufficient to change the phenotype of TSD-fibroblasts.

The major challenges in TSD gene therapy are the choice of the vector and delivery method of therapeutic genes in order to overcome the blood-brain barrier along with minimal side effects (Kyrkanides et al., 2005). Martino et al. (2005) proposed an *in vivo* gene transfer strategy for the production and distribution of the *HEXA* gene in the CNS in TSD model animals. A replication-defective herpes simplex virus type 1 (HSV-1) encoding *HEXA* gene cDNA was made. HSV-1 is able to infect various types of non-dividing cells, including neurons, and is transferred in a retrograde fashion to motor and/or sensory neuron bodies after peripheral inoculation (Wolfe et al., 1999). It was shown that the injection of HSV-1-HEXA into the inner capsule of the left cerebral hemisphere of TSD model mice restored HexA activity. GM2 ganglioside accumulation disappeared both in the injected and in the control (right) hemispheres, as well as in the cerebellum and spinal cord of the studied animals within a month after the injection (Martino et al., 2005). Thus when the viral vector is directly delivered to the brain of laboratory animals, a high efficiency of cell transduction is shown. However, due to the large size of a human brain, this approach has limitations for the uniform distribution of the viral vector throughout the central nervous system and would require a large number of injections.

Great progress has been achieved in developing approaches for GM2 gangliosidoses gene therapy using adeno-associated virus (AAV)-based vectors (Gray-Edwards et al., 2018). Intracranial administration of recombinant AAV (rAAV) serotypes 2/1 or 2/2 encoding the *HEXA* and *HEXB* gene cDNA to SD model mice led to a wide spread of HexA in the nervous system without apparent cytotoxicity associated with rAAV use and an increase in mouse survival rate (Cachon-Gonzalez et al., 2006, 2012). Defects in myelination occur at an early age and so there are time limits within which gene therapy can lead to positive results and slow the progression of neurological worsening (Cachon-Gonzalez et al., 2014).

Adeno-associated virus-based vectors are limited in their capacity (from 2.1 to 4.5 kb) and cannot carry the cDNA of both *HEXA* and *HEXB* genes, and the efficiency of co-transduction is significantly less than transduction with a single construct. This is a limiting factor in the application of these vectors since the effective recovery of the secretion of the absent heterodimeric HexA isoenzyme requires the expression of both subunits, α and β (Tropak et al., 2016). Tropak et al. (2016) designed the self-complementary AAV9.47 encoding a hybrid μ subunit (scAAV9.47-HEXM) and showed that intracranial injection of scAAV9.47-HEXM decreased GM2 ganglioside accumulation in the brain of TSD model mice. Intravenous administration of scAAV9.47-HEXM to newborn TSD model mice showed a long-term decrease in GM2 ganglioside accumulation in the CNS and a decreased distribution of this vector in the liver compared to AAV9, AAVrh10 or AAVrh8 (Karumuthil-Melethil et al., 2016). Intravenous injection of scAAV9.47-HEXM results in effective transduction of CNS cells and an 2.5-fold increase in survival rate of newborn SD model mice compared with the control group (Osmon et al., 2016).

The therapeutic efficacy of AAVrh8-based gene therapy on the Jacob sheep TSD model has been studied. Sheep aged 2–4 months received intracranial injections of only AAVrh8 encoding α subunit (AAVrh8-HEXA), or two vectors encoding α or β subunit (AAVrh8-HEXA + AAVrh8-HEXB). It was shown that all sheep after AAV injection had a delay in the appearance of symptoms and/or a decrease in the acquired symptoms of the disease. When AAVrh8-HEXA + AAVrh8-HEXB were injected, an excellent distribution of HexA in the sheep brain was noted, unlike the injection of AAVrh8-HEXA. However, HexA distribution in the sheep spinal cord was low in all groups and a decrease in the activation and proliferation of microglia in the sheep brain after gene therapy was noted (Gray-Edwards et al., 2018). The data were confirmed by studies with SD cats that demonstrated safety and a wide spread of Hex in the CNS, after intracranial injection of AAVrh8 encoding the species-specific α and β Hex subunits (Bradbury et al., 2013).

A study of the safety of AAVrh8 encoding the α and β Hex subunit of normal cynomolgus macaques (cm) showed that dyskinesia, ataxia, and loss of dexterity developed in most of the monkeys with intracranial injection of AAVrh8-cmHexα/β (Golebiowski et al., 2017). Animals that received a high dose of AAVrh8-cmHexα/β eventually became apathetic. The time of symptom onset depended on the dose, with the highest dose causing symptoms within a month after the infusion. Histological analyses showed severe necrosis of white and gray matter along the injection pathway, the reactive vasculature and the presence of neurons with granular eosinophilic material. Despite neurotoxicity, a sharp increase in Hex activity was noted in the thalamus (Golebiowski et al., 2017). The authors suggested that severe neurotoxicity may be associated with Hex overexpression. The data about TSD gene therapy are generalized in Table 1.

**Table 1:** *In vivo* investigations of Tay-Sachs disease gene therapy effectiveness.

Vector	Gene	Model	Injection method	Result	Reference
Recombinant adenovirus	*HEXA HEXB*	*HEXA* knockout mice	Intravenously	HexA secretion in the serum and enzymatic activity restoration in peripheral tissues. Preferential transduction of liver cells is observed	Guidotti et al., 1999
HSV-1	*HEXA*	TSD model mice	Intracranial to the inner capsule of the left cerebral hemisphere	High efficiency of cell transduction, HexA activity restoration and removal of GM2 ganglioside accumulation in both hemispheres of the brain	Martino et al., 2005
rAAV 2/1 or 2/2	*HEXA HEXB*	SD model mice	Intracranial	Wide spread of HexA in the nervous system and increased survival rate	Cachon-Gonzalez et al., 2006, 2012, 2014
scAAV9.47	*HEXM*	TSD model mice	Intracranial	Reduction of GM2 accumulation in the brain of mice	Tropak et al., 2016
scAAV9.47	*HEXM*	TSD model newborn mice	Intravenously	Long-term decrease in GM2 ganglioside accumulation in the CNS and decrease in biodistribution of the vector in the liver	Karumuthil-Melethil et al., 2016
scAAV9.47	*HEXM*	SD model newborn mice	Intravenously	Reduction of GM2 accumulation in the CNS, an 2.5-fold increase in the survival rate of mice	Osmon et al., 2016
AAVrh8	*HEXA HEXB*	TSD Jacob sheep	Intracranial	Delay in the symptom manifestation and/or a decrease in the acquired symptoms, decrease in the activation and proliferation of microglia in the sheep brain. Low HexA distribution in the spinal cord was noted	Gray-Edwards et al., 2018
AAVrh8	*HEXA HEXB*	SD cats	Intracranial	Safety and wide spread of Hex in the CNS	Bradbury et al., 2013
AAVrh8	*HEXA HEXB*	Normal cynomolgus macaques	Intracranial	A sharp increase in Hex activity. The development of neurotoxicity, presumably due to Hex overexpression	Golebiowski et al., 2017

## Genetically Modified Multipotent Cells

The transplantation of *ex vivo* modified multipotent neural cells (MNCs) in the CNS is another therapeutic strategy. An MNC line with human *HEXA* gene overexpression (MNCs-HEXA) has been produced by retroviral transduction Lacorazza et al. (1996). MNCs-HEXA stably secreted the biologically active HexA enzyme and cross-corrected the metabolic defect in TSD patient-derived fibroblast culture *in vitro*. Intracranial injection of MNCs-HEXA to mice resulted in expression of a significant quantity of the human HexA subunit transcript and active HexA enzyme production (Lacorazza et al., 1996). It has been shown that transduction of stromal cells obtained from the bone marrow of TSD model mice, with retrovirus encoding *HEXA* gene cDNA, results in an increase in secretion of the active HexA enzyme capable of hydrolyzing GM2 ganglioside (Martino et al., 2002a).

## Treatment Strategies for Lysosomal Storage Disorders

Currently, the number of available treatments for patients with LSDs is increasing. For example, BMT (Lange et al., 2006; Rovelli, 2008), SRT (Coutinho et al., 2016), and ERT (Li, 2018) are used for therapy of Gaucher disease, Fabry disease, mucopolysaccharidoses (MPS), Pompe disease, Niemann-Pick disease, etc. ERT and SRT methods are approved for these diseases in Europe, United States, and other countries (Beck, 2018). The previously described drug Zavesca^®^ is also used for the treatment of Niemann-Pick disease type C (Hassan et al., 2018; Pineda et al., 2018) and GM1-gangliosidosis (Deodato et al., 2017). It is worth mentioning that, similarly to TSD, early diagnosis is necessary for successful LSD therapy in order to prevent organ damage that aggravates the disease progression (Wasserstein et al., 2018).

Lifelong prescription of ERT with recombinant glucocerebrosidase showed good results in the treatment of Gaucher disease. ERT stops the main clinical manifestations of the disease, improves the quality of life of patients and has no pronounced adverse effects (Zimran et al., 2018). However, for some LSDs, such as MPSI, II and VI, Pompe disease and diseases with CNS damage, ERT is not effective as the large molecules of recombinant enzymes are unable to penetrate damaged tissues to achieve therapeutic levels (Wraith, 2006; Begley et al., 2008; Parenti et al., 2013).

Non-TSD LSDs caused by missense mutations and small deletions without frameshift can be treated with the use of small molecules of pharmacological chaperones to increase active enzyme concentration (Pereira et al., 2018). If the mutation does not affect the active or binding site of the enzyme and only leads to disruption of the protein conformation then pharmacological chaperones can be used as protein stabilizers to form a stable structure and maintain catalytic activity (Parenti, 2009). The molecular chaperones thereby increase the intracellular pool of active enzymes and can partially restore metabolic homeostasis. The application of this approach is under investigation for Gaucher disease (Goddard-Borger et al., 2012), Fabry disease (Kato et al., 2010), Pompe disease (Porto et al., 2009), and Krabbe disease (Berardi et al., 2014).

For many LSDs gene therapy approaches [comprehensively discussed in the review (Biffi, 2016)] as well as genome editing techniques using zinc-finger nucleases (ZFN) for MPSI and MPSII (Harmatz et al., 2018) are being actively explored. Improvement of LSD treatment methods, in particular, using pharmacological chaperones and genome editing technologies can also contribute to the development of new approaches to TSD treatment which is important since the current approved treatment options for this disease have low efficiency.

## Conclusion

To achieve a therapeutic effect in the treatment of TSD the production and distribution of the absent HexA enzyme in CNS is required. SRT, ERT, and BMT showed low efficacy to prevent neurodegeneration in the CNS although these methods can partially restore HexA activity and reduce GM2 ganglioside accumulation in cells (Table 2). It is important to remember that in order to achieve the maximum therapeutic effect, it is necessary to start TSD therapy from the time of its early manifestations, since myelination defects appear at early stages and are aggravated with time.

**Table 2:** Efficacy of various therapeutic approaches for TSD treatment in pre-clinical and clinical trials.

Therapeutic approach	Small animal models	Large animal models	Clinical trials/case reports in TSD patients
Substrate reduction therapy	Miglustat was shown to prevent the GM2 ganglioside accumulation in the brain of TSD model mice (Platt et al., 1997; Bembi et al., 2006)	N/A	Use of miglustat did not stop the neurologic dysfunction progression (NCT00672022) (Maegawa et al., 2009). SRT is recommended for macrocephaly prevention (Bembi et al., 2006)
Enzyme replacement therapy	Improvement of motor function and increased survival rate in SD model mice (Matsuoka et al., 2011; Tsuji et al., 2011)	N/A	No registered clinical trials available
Bone marrow transplantation	Increased from 4.5 to 8 months survival rate in SD model mice, improvement of neurological manifestations (Norflus et al., 1998; Wada et al., 2000)	N/A	Only case reports available: HexA activity increase. Neurologic dysfunction progression was not stopped (Jacobs et al., 2005; Stepien et al., 2017)
Gene therapy (see Table 1 for more detailed information)	HexA activity restoration and removal of GM2 ganglioside accumulation in CNS and increased survival rate in SD or TSD model mice	Safety and wide spread of HexA in the CNS in SD cats. TSD Jacob sheep delay in the symptom manifestation and inflammation reduction in CNS were observed. In normal cynomolgus macaques the development of neurotoxicity in response to gene therapy drug injection is shown	No registered clinical trials available
Administration of multipotent cells genetically modified with HexA	HexA activity increase after injection to mice (Lacorazza et al., 1996)	N/A	No registered clinical trials available

Currently there are methods of TSD gene therapy being developed using viral vectors for the delivery of cDNA of encoding α and β HexA subunit genes. In humans, it is necessary for viral vectors to successfully cross the blood-brain barrier since the injection of genetic constructs directly into the CNS is not feasible due to the large size of the human brain. In addition, severe neurotoxic effects due to HexA overexpression in case of direct viral delivery to the brain of cynomolgus macaques gives rise to concern.

Of particular interest are studies using the scAAV9.47 vector encoding the *HEXM* gene of the hybrid μ subunit that contains the α subunit active site, the stable β subunit interface, and also the unique regions in each subunit that are required for interaction with GM2A. This vector is able to cross the blood-brain barrier and the *HEXM* gene circumvents the capacity limitation of AAV vectors. However, studies of the efficacy of this viral construction are currently limited to *in vivo* experiments in TSD or SD model mice.

Further investigation of the therapeutic potential of genetically modified stem cells is important, since in addition to restoring the activity of the deficient enzyme, these cells can have a neuroprotective effect to limit the degenerative processes that are observed in TSD patients. This, combined with stem cells can prevent the activation of microglia guarding against further neurodegeneration.

## Author Contributions

VS and AR conceived the idea. VS wrote the manuscript and made the tables. AS and DC collected the data of TSD gene therapy. KK created the figure. LC and AR edited the manuscript and tables. All authors contributed to read and commented on the manuscript.

## Conflict of Interest Statement

The authors declare that the research was conducted in the absence of any commercial or financial relationships that could be construed as a potential conflict of interest.
